# Structural characterization, antioxidant and anti-uropathogenic potential of biogenic silver nanoparticles using brown seaweed *Turbinaria ornata*

**DOI:** 10.3389/fmicb.2023.1072043

**Published:** 2023-09-01

**Authors:** C. T. Dhanya Raj, Krishnan Muthukumar, Hans Uwe Dahms, Rathinam Arthur James, Surabhi Kandaswamy

**Affiliations:** ^1^Department of Marine Science, Bharathidasan University, Tiruchirappalli, Tamil Nadu, India; ^2^Department of Petrochemical Technology, Bharathidasan Institute of Technology, Anna University, Tiruchirappalli, Tamil Nadu, India; ^3^Department of Biomedical Science and Environmental Biology, Kaohsiung Medical University (KMU), Kaohsiung, Taiwan, China; ^4^Research Centre for Precision Environmental Medicine, Kaohsiung Medical University, Kaohsiung, Taiwan, China; ^5^Department of Marine Biotechnology and Resources, National Sun Yat-sen University, Kaohsiung, Taiwan, China; ^6^Manchester Centre for Genomic Medicine, School of Biological Sciences, Faculty of Biology, Medicine and Health, The University of Manchester, Manchester, United Kingdom; ^7^School of Pharmacy and Biomedical Sciences, University of Central Lancashire, Preston, United Kingdom

**Keywords:** silver nanoparticles, uropathogens, urinary tract infections, *T. ornata*, antioxidant, anti-uropathogenic

## Abstract

Alternative treatment strategies for urinary tract infections (UTIs) are becoming more necessary due to increasing drug resistance patterns in uropathogens. Nanoparticle-based therapeutics is emerging as a way to treat UTIs. In the present study, using *Turbinaria ornata* extract, silver nanoparticles (AgNPs) were synthesized, characterized, and their anti-uropathogenic activity was evaluated. The stability and formation of synthesized To-AgNPs were confirmed by UV-visible spectroscopy, FTIR, XRD, SEM, and DLS. An FTIR spectrum confirmed the presence of seaweed functional groups in To-AgNPs, a XRD analysis confirmed their crystalline nature, and SEM imaging confirmed their spherical nature with an average size of 73.98 nm with diameters ranging from 64.67 to 81.28 nm. This was confirmed by TEM results. DLS determined that the cumulant hydrodynamic diameter of To-AgNPs was 128.3 nm with a PdI of 0.313 and the zeta potential value were found to be –63.3 mV which indicates the To-AgNPs are negatively charged and more stable. DPPH assays were used to assess the antioxidant activity of biosynthesized To-AgNPs, while an agar well diffusion method was used to test the antibacterial activity against uropathogens, including *Staphylococcus aureus, Escherichia coli, Pseudomonas aeruginosa, Enterococcus faecalis*, and *Klebsiella pneumoniae*. The To-AgNPs showed the highest susceptibility to *S. aureus* (15.75 ± 0.35 mm) and *E. coli* (15 ± 0.7 mm) with MIC values of 0.0625 and 0.125 mg/ml, respectively in macro broth dilution method and observed considerable membrane damage under CLSM and SEM. To-AgNPs displayed stronger antioxidant and antimicrobial activity, suggesting they may be developed as a new class of antimicrobial agents for treating UTIs.

## Graphical Abstract

Antibacterial activity of *T. ornata*, To-AgNps and Cefotaxime (30 mcg) against selected uropathogens. 
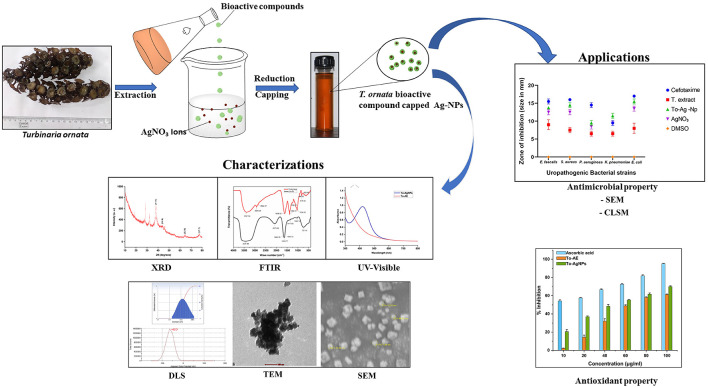

## 1. Introduction

Urinary tract infections (UTIs) are the second most important clinical diseases that account for up to 40% of nosocomial infections. Each year, about 150 million people are affected globally, resulting in an economic and public health burden of about $3.5 billion annually in the United States (Flores-Mireles et al., 2015). Among elderly men, women of any age, and infant boys, UTIs account for significant morbidity. Women are more susceptible to UTIs than men (Medina and Castillo-Pino, 2019). The most common uropathogens are *Escherichia coli*, *Enterococcus* spp., *Pseudomonas aeruginosa*, *Klebsiella pneumoniae*, *Staphylococcus* spp., *Proteus mirabilis*, and *Candida* spp. Most UTIs (80%) are caused by *E*. *coli*. However, patients with complicated UTIs have a higher rate of non-*E*. *coli* infections (44–72%) (Tabibian et al., 2008; Brumbaugh and Mobley, 2012). The severity of UTIs varies from a mild innocuous infection to acute sepsis with mortality rates between 20 and 40% (Wagenlehner et al., 2013; Das, 2020). Additional complications are kidney infections with a risk of permanent kidney damage caused by poorly treated or untreated UTIs. People with recurrent UTIs often experience depression and anxiety symptoms.

Initial broad-spectrum antibiotics, such as penicillins, cephalosporins, beta-lactams, carbapenems, and fluoroquinolones are effective in treating recurrent UTIs. However, inappropriate selection of antibiotics or long-term usage leads to antibiotic resistance (Sánchez et al., 2021). Drug-resistant bacteria pose a serious threat that necessitates the development of alternative antimicrobials. With the advances in nanotechnology, nanomaterials have shown great promise in the field of biomedicine. In recent years, many kinds of metallic nanoparticles have been developed and evaluated for their antibacterial properties, but silver nanoparticles (AgNPs) are the most appealing and ideally suited for biomedical applications due to their unique physical properties, high photo thermal effect and are effective in killing bacteria. Anticancer, antimicrobial, antioxidant, and anti-inflammatory are some of their medicinal uses. The benefits of AgNPs include they are less toxic, they do not alter the host cellular structure, and they cannot cause microbial resistance (Franci et al., 2015). In spite of the widespread knowledge of AgNPs’ antibacterial properties, the mechanisms of their action remain largely obscure. AgNPs exhibit high antimicrobial activity because of their small size and large surface area; AgNPs get incorporated into the cell membrane, alter the membrane potential, increase permeability to ions, or inhibit enzymes that control cell division (Bruna et al., 2021). They also modulate cellular signaling by dephosphorylating putative key peptide substrates on tyrosine residues (Bouqellah et al., 2019). Many researchers have determined that AgNPs can be used as antimicrobial agents against *Escherichia coli, Klebsiella pneumoniae, Staphylococcus aureus*, and *Pseudomonas aeruginosa* (Li et al., 2011; Salomoni et al., 2017; Loo et al., 2018).

In recent years, green synthesis of AgNPs has gradually replaced physiochemical methods due to issues related to the consumption of large amounts of energy, the release of toxic and harmful chemicals, and the need for complex equipment and conditions for NP synthesis. Green synthesis involves using natural and environmentally friendly materials (microorganisms, phytochemicals, and antioxidants) as reducing agents and capping agents, thereby reducing energy consumption, avoiding the usage of high-pressure and toxic reagents, cost efficiency, and large-scale production (Guan et al., 2022). The physicochemical properties, shape, size, and stabilization of AgNPs can be modified by capping agents. Therefore, choosing an appropriate capping agent will strengthen and improve the interaction of the nanoparticles (NPs) with the surroundings and improve cytotoxicity and biocompatibility. Several studies have shown that seaweeds are rich sources of secondary metabolites which can suitably act as capping and reducing agents in AgNP synthesis (Kannan et al., 2013; de Aragão et al., 2019; Bhuyar et al., 2020). Secondary metabolites also have a range of beneficial attributes such as antimicrobial, diuretic, antioxidant, analgesic, anti-inflammatory, anti-cancer, anti-apoptotic, enzyme inhibitory, and antiurolithiatic properties.

*Turbinaria ornata* (*T. ornata*) is a brown alga that belongs to the Sargassaceae family, of the class Phaeophyceae and is found in tropical and subtropical regions of the Western and Central Pacific, as well as the Indian Ocean. They occur in small clusters closely related to the fissures of basalt rocks in high wave action regions, apart from the fissures of coral heads at 20–30 m. This alga’s morphological characteristics enable it to survive in challenging ecological conditions (Remya et al., 2022). *T. ornata* is rich in bioactive compounds including saponins, fucoxanthin, terpene flavonoids, phenols, proteins, sulfates, polysaccharides, fucosterol, polyphenols, and alkaloids (Deepak et al., 2017). These bioactive compounds can reduce silver ions to silver and thus stabilize NPs (Khan et al., 2022). According to published records, it has extensive biological properties such as anti-coagulant, antioxidant (Arivuselvan et al., 2011; Alharbi et al., 2020), antitumor activity (Remya et al., 2017), anti-inflammatory (Ananthi et al., 2010; Subash et al., 2016), anti-cancer (Bharath et al., 2021), antibacterial (Ward and Deyab, 2016), anti-diabetic (Unnikrishnan et al., 2014), and wound-healing (Senthil and Annappan, 2013; Shaibi et al., 2022).

A few studies have examined the antibacterial effects of various nanoparticles biosynthesized from *T. ornata*, such as tin oxide (Sn-O_2_) nanoparticles (Sunny et al., 2022), magnesium hydroxide (Mg(OH)_2_) nanoparticles (Govindaraju et al., 2020), and gold (Au) and Ag-NPs (Kayalvizhi et al., 2014). Most metal nanoparticles may produce toxic ions, cytokines, and reactive oxygen species (ROS) that are toxic to healthy and infected cells. The advantages of AgNPs over other nanoparticles include their cost-effectiveness, low toxicity, high efficiency, low maintenance, ease of use, and biocompatibility. Additionally, bioactive compounds in *T. ornata* extract bind and stabilize AgNPs, making them more effective against microbes. Several studies have demonstrated the antibacterial effect of AgNPs synthesized from *T. ornata* against biofilm microbes (Krishnan et al., 2015) and water-contaminating bacteria (Anuluxan et al., 2022). To our knowledge, there have been no reports on *T. ornata*-mediated AgNPs as an uropathogen target. Hence, this study aims to investigate the antiuropathogenic activity of the *T. ornata* extract (To-AE) and its biosynthesized AgNPs. The mechanism involved in *in vitro* antibacterial activity was further investigated using scanning electron microscopy (SEM) and confocal laser scanning microscopy (CLSM).

## 2. Materials and methods

### 2.1. Chemicals and materials

Analytical grade chemicals with maximum purity were used in this study. Silver nitrate (AgNO_3_) and bacterial culture media were purchased from Himedia Laboratories Ltd., India. Uropathogens responsible for UTIs such as *S. aureus*, *K. pneumoniae*, *E. coli*, *P. aeruginosa*, and *E. faecalis*, were granted by the K.A.P. Viswanatham Medical College, Tiruchirappalli – 620 001, Tamil Nadu.

### 2.2. Seaweed collection and processing

**FIGURE 1:**
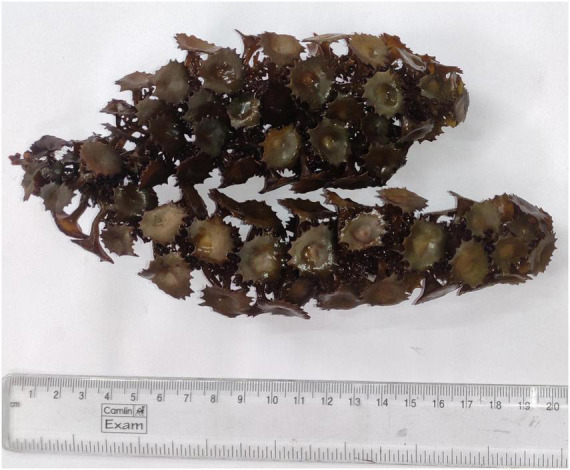
Brown seaweed *Turbinaria ornata* collected from Mandapam coastal area of Tutucorin, Tamil Nadu, India.

Fresh *T. ornata* seaweeds (Figure 1) were collected from the Mandapam coastal area of the Gulf of Mannar located at the Bay of Bengal region (78°8’ E latitude and 9°17’ N longitude) of Tutucorin, Tamil Nadu, India. The seaweed samples were hand-picked and washed thoroughly in filtered seawater, tap water and distilled water to clean up adhering epiphytes, debris, and other impurities. Seaweed thalli with distinct morphology were transported immediately in separate polyethylene bags to the laboratory and shade dried for 3–5 days at room temperature (Harinee et al., 2019). The dried seaweed was then ground to fine powder with electric blender and stored in air tight container for further use.

### 2.3. Preparation of seaweed extract

Aqueous seaweed extract was made by adding 50 g of dried seaweed powder to 400 ml distilled water and heated to boiling until it was reduced to half its original volume. Once it cooled, the extract was filtered using Whatman grade 42 filter paper (Whatman, GE Healthcare, UK) and stored at 4°C for further studies (Harinee et al., 2019; Bhuyar et al., 2020).

### 2.4. Biosynthesis of To-AgNPs

Seaweed extract-mediated AgNPs were synthesized according to Azeem et al. (2021) with modifications. *T. ornata* extract (To-AE) (5 ml) was stirred with 95 ml of 1 mM AgNo_3_ solution after adjusting the pH to 10 by adding 1 M NaOH. At alkaline pH the AgNPs formation gets accelerated. Upon completion of the reaction, a change in color was observed. The product was centrifuged at 10,000 rpm for 20 min. The pellets were collected and washed with distilled water to obtain pure NPs.

### 2.5. Characterization of To-AgNPs

The characterization of To-AgNPs is critical for elucidating their functional properties because their physicochemical properties have profound effects on behavior, biodistribution, safety, and efficacy. An array of analytical techniques was employed for characterizing the samples, including UV-visible spectroscopy, Fourier transform infrared spectroscopy (FTIR), X-ray diffractometry (XRD), SEM, and dynamic light scattering (DLS) (Chandraker et al., 2022a,b). A UV-vis spectrophotometer (Lambda 35, Perkin Elmer) was used to confirm the NPs formation. The absorption spectrum of the AgNPs was recorded over a wavelength range of 300–800 nm. The type of seaweed biomolecules responsible for the reduction of AgNPs was determined by FTIR spectroscopy. Surface capping of the To-AgNPs by phytochemical constituents of seaweed extract was also confirmed by FTIR (Spectrum RX-I, Perkin Elmer) with a wavelength of 4,000–400 cm^–1^. The size and morphological characteristics of the NPs were determined by SEM (EVO-18, Carl Zeiss) and TEM (FEI-TECNAI, G2-20 Twin microscope). XRD (Rigaku ULTIMA III) was used to determine the crystalline nature and lattice structure of the synthesized NPs, with Cu- kα anode radiation (λ = 1.54056 Å) operated at a voltage of 45 kV and a current of 30 mA, over a 2*θ* collection range of 20–80° with a step size of 0.02° and scan rate of 4°/min. To-AgNPs particle size distribution and zeta potential were determined using DLS (Micromeritics, Nano Plus).

### 2.6. Antioxidant activity

DPPH radical scavenging activity of To-AE and To-AgNPs was evaluated by the method of Keshari et al. (2018). Briefly, 3 ml samples at different concentrations (10, 20, 40, 60, 80, and 100 μg/ml) were mixed with 1 ml of 0.1 mM DPPH in methanol and incubated for 30 min in the dark. The positive control was ascorbic acid. The absorbance of the samples and control was recorded at 517 nm spectroscopically and radical scavenging activity was calculated using the formula below.


(1) %Radicalscavengingactivity=[(A0-A1)/A0]×100

A0 and A1 are the absorbance of the control and experimental samples, respectively.

### 2.7. Anti-uropathogenic activity

The anti-uropathogenic activity of To-AE and To-AgNPs was studied by the well diffusion method (Faraja et al., 2018). Overnight cultures of UTIs causing bacteria, such as *E. coli*, *E. faecalis*, *K. pneumoniae, S. aureus*, and *P. aeruginosa* were used for the study. Mûller Hinton agar was prepared and poured into 4 petri plates and allowed to cool down. Once the medium got solidified, agar plates were swabbed with the inocula of the test pathogens. Wells (6 mm diameter) were cut out of the agar, and 50 and 100 μl of To-AE and the To-AgNPs were placed into each well and incubated at 37°C for 24 h. At the end of the incubation the diameters of the clear zone of inhibition of growth were measured.

### 2.8. Minimum inhibitory concentration (MIC) and minimum bactericidal concentration (MBC) assay

The MIC and MBC of To-AgNPs were determined according to CLSI guidelines (Clinical and Laboratory Standards Institute [CLSI], 2015). MIC tests were performed as a standard macro broth dilution method, whereas MBC tests were carried out on the MHA plates. Bacteria for the experiment were 10^6^ CFU/ml, and two fold serial dilutions of To-AgNPs were prepared by mixing 1 ml of the working solution with 1 ml of bacterial inoculum in MHB. The concentration gradients of To-AgNPs were 2–0.0156 mg/ml. Additionally, a negative control (medium only) and a positive control (medium and bacterial inoculum) were maintained. Then the tubes were incubated for 24 h at 37°C. A MIC value was determined with the lowest concentration of To-AgNPs that inhibited bacterial growth. The MBC test was performed by plating the suspension from each tube which showed no visible bacterial growth, onto a MHA plate which was then incubated for 24 h. A MBC value was taken as the lowest concentration to kill the bacteria.

### 2.9. Confocal laser scanning microscopy (CLSM)

Confocal microscopy (Carl Zeiss, LSM710, Jena, Germany) was used to examine antimicrobial activity of To-AgNP against *S. aureus* and *E. coli* (Henciya et al., 2020). To-AgNPs were used at a concentration of 0.125 mg/mL for *E. coli* and 0.0625 mg/ml for *S. aureus* based on MIC results. Cultures without treatment were used as controls. Acridine orange and ethidium bromide (AO-EtBr) was used to stain the treated and untreated bacterial cells, and live and dead cells were observed under the confocal microscope.

### 2.10. Scanning electron microscopy (SEM)

The visualization of impact of To-AgNPs on the structure and morphology of *S. aureus* and *E. coli* cells was performed by SEM microscopy according to the following procedure (Zawadzka et al., 2016). Overnight cultures of treated and untreated samples were centrifuged at 6000 rpm for 15 min and pellets were collected thereafter. After being washed thrice with sterile PBS the pellets were suspended in cold glutaraldehyde and incubated for 2 h at 4°C. Bacterial cells were then washed three times with sodium buffer and dehydrated with a series of ethanol solutions (30-100%). A subsequent SEM examination was carried out by coating washed bacterial cells on SEM stubs (EVO-18, Carl Zeiss, Germany).

### 2.11. Statistical analysis

A statistical analysis of the data was performed using GraphPad Prism Software (Version 9.0.0). The normality distribution of the antioxidant activity and antimicrobial activity assay data was examined using the Shapiro–Wilk test. One-way ANOVA was used to analyze data, which passed the Shapiro–Wilk test. The Tukey–Kramer *post-hoc* test was used to further analyze the data for multiple comparisons. All the experiments were performed in triplicates and the data obtained were reported as means ± standard deviation, and statistically significant differences between the treatment and control groups were considered at a *P*-value ≤ 0.05.

## 3. Results

### 3.1. Synthesis and characterization of AgNPs

The aqueous extracts of seaweed *T. ornata* were obtained using the hot extraction method. A color change from dark yellow to brown was observed after the addition of To-AE to AgNO_3_ solution, which indicated the reduction of silver ions (Ag^+^) to silver (Ag^0^) upon excitation of surface plasmon resonance (SPR). To obtain pure NPs, the solution was centrifuged at 10,000 rpm for 20 min, and pellets were washed with distilled water.

### 3.2. UV-visible spectroscopy

**FIGURE 2:**
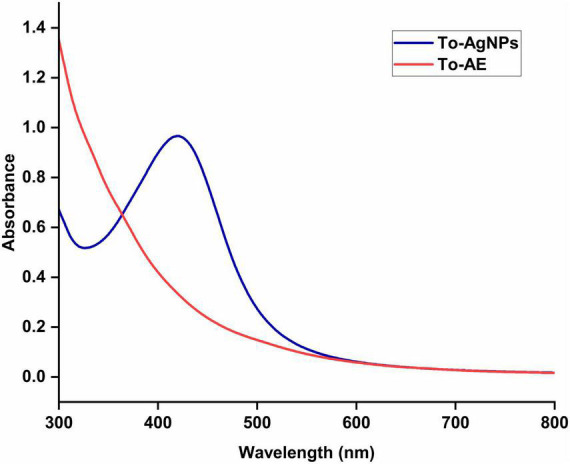
UV-Visible spectra of aqueous extract and biofabricated AgNPs of *T. ornata.*

To-AgNPs formation was confirmed using a UV–visible spectrophotometer. The UV absorption band for the To-AgNPs, at 420 nm, is shown in Figure 2.

### 3.3. FT–IR analysis

**FIGURE 3:**
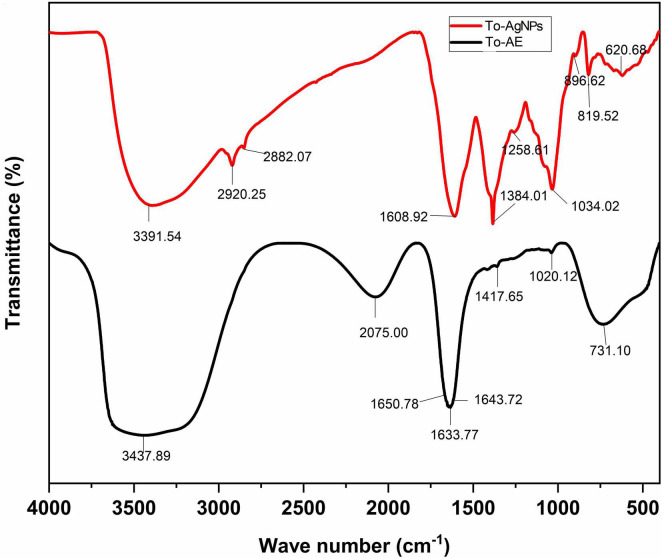
FT-IR spectra of *T. ornata* aqueous extract and To-AgNPs.

The biomolecule nano-capping of the extract around the synthesized To-AgNPs confirmed by FT-IR spectroscopy led to greater stability of the synthesized NPs. To-AE was shown to contain functional groups such as alkanes, methylene, amines, and carboxylic acids that are potential reducing agents for the synthesis of AgNPs (Cho et al., 2005). The FTIR analysis of To-AE indicated the presence of carboxylic acids and amines. Figure 3 shows the molecular arrangement of various functional groups in the To-AE and the powdered To-AgNPs. A comparison of transmission peaks revealed that suppressed/increased peaks in colloidal solutions were due to metal NPs bound to bioorganic molecules. The shifted peak at 3,439.87 represents O–H stretching modes formed by flavonoid and phenolic groups in the phytoconstituents. The band at 2,075.00 cm^–1^ confirmed the presence of nitrile carbons and C=O (1,650.78, 1,643.72, and 1,633.77 cm^–1^) was due to a free carboxylic acid group. Another band at 1022.43 cm^–1^ can be ascribed to the C–OH vibrations of alcohol groups in the To-AE. The FT-IR spectra of To-AgNPs also indicated the presence of functional groups like phenolic O–H (3,391.54 cm^–1^), nitrile carbons (2,882.07 cm^–1^), and C=O (1,608.92 cm^–1^).

### 3.4. X-ray diffraction analysis

**FIGURE 4:**
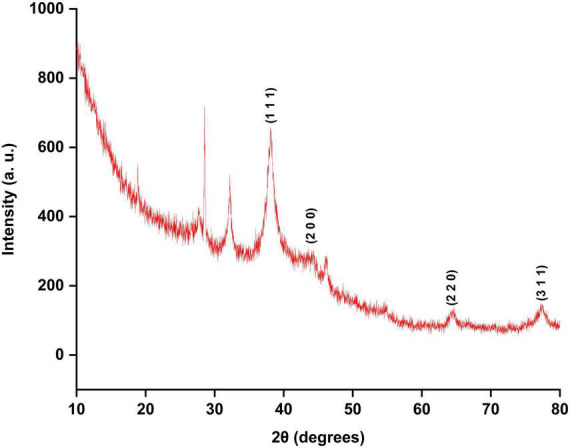
XRD pattern of To-AgNPs.

The intensities of diffracted X-rays were recorded from 20° to 80°. Four Bragg reflections at 38.07°, 43.97°, 64.36°, and 77.32° represented the lattice planes of (1 1 1), (2 0 0), (2 2 0), and (3 1 1), respectively (Figure 4), which confirmed the cubic structure of AgNPs. According to the Joint Committee on Powder Diffraction Standards (JCPDS) – 040783, the XRD results verified that the To-AgNPs formed by seaweed extract reduction of Ag^+^ ions were crystalline (Chandraker et al., 2022c). Additionally, peaks were observed at 28.57° and 32.15°, suggesting that bio-organic crystallization occurred on AgNPs surfaces.

### 3.5. Morphological analysis by SEM and TEM

**FIGURE 5:**
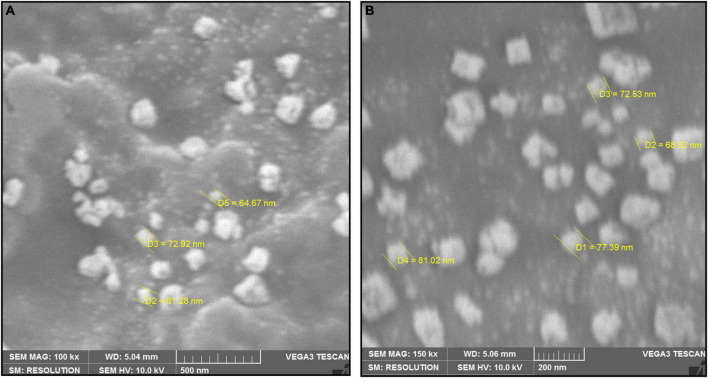
SEM image of synthesized To-AgNPs at two different magnificationsat two different magnifications, **(A)** 500 nm and **(B)** 200 nm.

**FIGURE 6:**
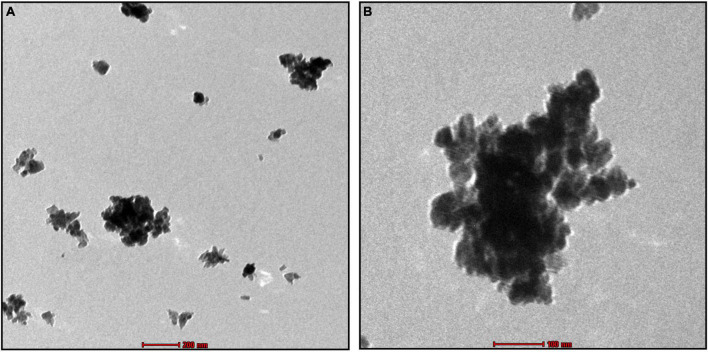
TEM image of synthesized To-AgNPs at two different magnifications at two different magnifications, **(A)** 500 nm and **(B)** 200 nm.

The SEM image provided an overview of the surface morphology, size and shape of the biosynthesized To-AgNPs (Merin et al., 2010). As shown in Figure 5, spherical and some irregular AgNPs with average size of 73.98 nm with diameters ranging from 64.67 nm to 81.28 nm were observed. This result strongly supported the notion that To-AE may act as reducing and capping agents in the production of To-AgNPs. As shown in TEM images (Figure 6), most of the particles are almost spherical, while others displaying irregular shapes, which is consistent with SEM results.

### 3.6. Dynamic light scattering (DLS)

**FIGURE 7:**
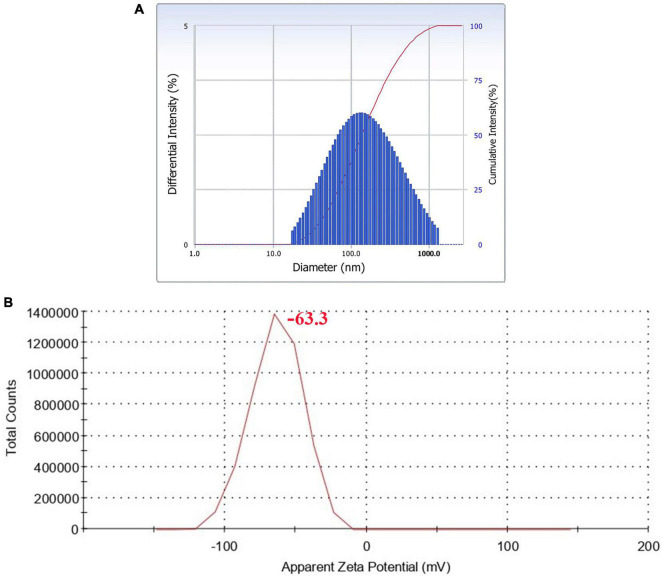
DLS histogram showing particle size distribution **(A)** and zeta potential **(B)** of synthesized To-AgNPs.

Hydrodynamic size distribution and the polydispersity index (PdI) of AgNPs were determined by DLS analysis (Adebayo-Tayo et al., 2019). The experiment also provided information about the population of particles in a short time. The results showed that the cumulant diameter of AgNPs at optimum conditions was 128.3 nm with a PdI of 0.313 (Figure 7A). Observation of the particle’s zeta potential reveals a sharp peak with a negative value (−63.3 mV), indicating the surface of the To-AgNPs was negatively charged and stable (Figure 7B).

### 3.7. Anti-oxidant activity

**FIGURE 8:**
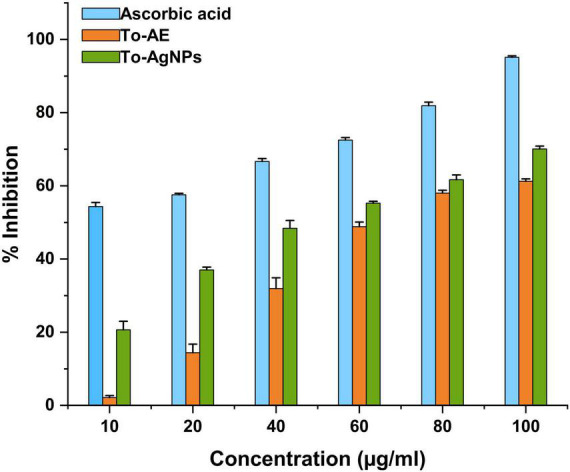
DPPH radical scavenging activity of To-AE, To-AgNPs, and ascorbic acid. Data were expressed as means ± standard deviation based on three independent replicates (*n* = 3).

**FIGURE 9:**
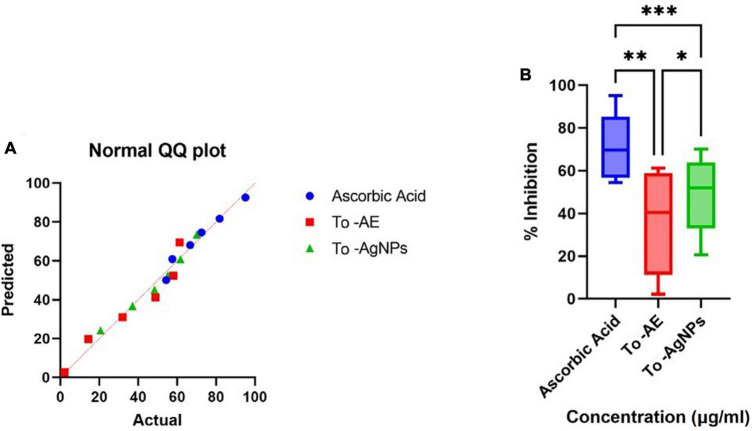
Statistical analysis of antioxidant activity of different groups **(A)** normality distribution of data, **(B)** one-way ANOVA between groups. (*P < 0.05, **P < 0.01, ***P < 0.001, All data were expressed as mean ± standard deviation based on three independent replicates, *n* = 3).

Ascorbic acid showed the highest antioxidant activity, followed by To-AgNPs and To-AE. Based on the concentrations, 100 μg/ml of ascorbic acid, To-AE, and To-AgNPs demonstrated excellent radical scavenging activity, 95.12, 61.27, and 70.09%, respectively (Figure 8). The Shapiro–Wilk normality test showed that the data had a significance of *P* < 0.05 (Figure 9A). A one-way ANOVA was applied to examine differences between the treatment groups (To-AE, To-AgNPs) and controls (ascorbic acid), which were statistically significant (DF = 5, *F* = 5.037, *P* = 0.0212). Based on a *Post-hoc* Tukey’s test, To-AE and To-AgNPs showed highly significant positive correlations with ascorbic acid, *P* = 0.0013 and *P* = 0.0007, respectively, and To-AgNPs showed a higher radical scavenging activity than To-AE (Figure 9B).

### 3.8. Anti-uropathogenic activity

**FIGURE 10:**
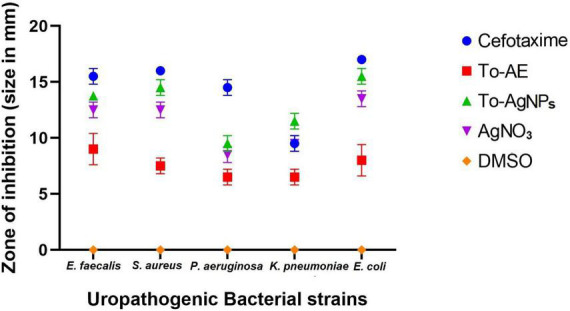
Anti-uropathogenic bacterial inhibition of different agents and controls. Error bar represents the mean ± standard deviation of three independent experiments.

Antimicrobial activity tests were performed against bacteria that cause UTIs including *K*. *pneumoniae*, *P. aeruginosa*, *E. faecalis*, *E. coli*, and *S. aureus.* As shown in Figure 10 and Supplementary Figure 1, To-AgNPs were more effective than AgNO_3_ solutions, and To-AE. Additionally, DMSO, as a negative control, did not show any inhibition zone. An antibiotic disk of 30 mcg Cefotaxime (CTX) was used as a positive control because it exhibited broad-spectrum antibacterial activity in most uropathogens. Among the selected uropathogens *S. aureus* (15.75 ± 0.35 mm) and *E. coli* (15 ± 0.7 mm) were more susceptible to To-AgNPs. A maximum inhibition zone was observed against Gram-positive *S. aureus*, while Gram-negative *P. aeruginosa* (9 ± 0.7 mm) had a minimum zone of inhibition.

**FIGURE 11:**
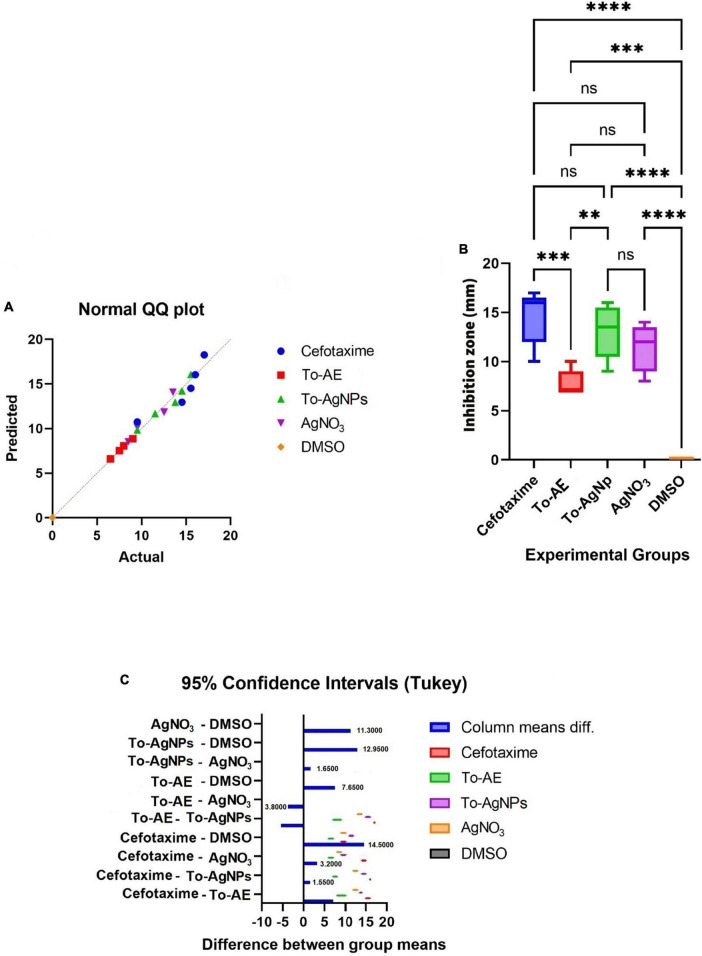
Statistical analysis of antimicrobial activity of different agents, **(A)** normality distribution of data, **(B)** one-way ANOVA between groups, **(C)** graphical display of Tukey’s multiple comparison tests for antimicrobial activity data. All data were expressed as mean ± standard deviation based on three independent replicates (*n* = 3), ** implies *P* < 0.01, *** implies *P* < 0.001, **** implies *P* < 0.0001, ns implies not significant.

As shown in Figure 11A, Shapiro–Wilk normality test results indicate statistical significance for normality distribution. *In vitro* antibacterial activity was compared with one-way ANOVA statistical analysis, and statistically significant results were obtained (*P* < 0.05). A Tukey’s test was conducted for multiple comparison of To-AE, To-AgNPs, AgNO_3_, positive control (Cefotaxime), and negative control (DMSO) inhibitory diameters for antimicrobial activity. In Figures 11B, C, it is seen that all treatment groups had significantly different antibacterial activities except for AgNO_3_, which was not significant when compared with Cefotaxime (mean difference (MD) = 3.2, *P* = 0.1198), To-AgNPs (MD = 1.65, *P* = 0.0076) and To-AE (MD = –3.8, *P* = 0.019), as well as Cefotaxime with To-AgNPs also not significant (MD = 1.55, *P* = 0.6588). Based on the ANOVA results, it can be concluded that To-AgNPs had significantly higher antibacterial activity than all other groups except Cefotaxime. Cefotaxime serves as a standard to correlate the lead agent between the groups.

### 3.9. MIC and MBC determination

To-AgNPs showed potent activity against *S. aureus*, *E coli, K. pneumoniae*, and *E. faecalis* with MIC values 0.0625, 0.125, 0.25, and 0.25 mg/ml, respectively. On the other hand, inhibitory activity was not detected against *P. aeruginosa*. The MBC against *S. aureus*, *E. coli, K. pneumoniae*, and *E. faecalis*, 0.125, 0.25, 0.5, 0.5 mg/mL, respectively. These results showed that the To-AgNPs were highly effective against *S. aureus* and *E. coli*.

### 3.10. Confocal laser scanning microscopy (CLSM)

**FIGURE 12:**
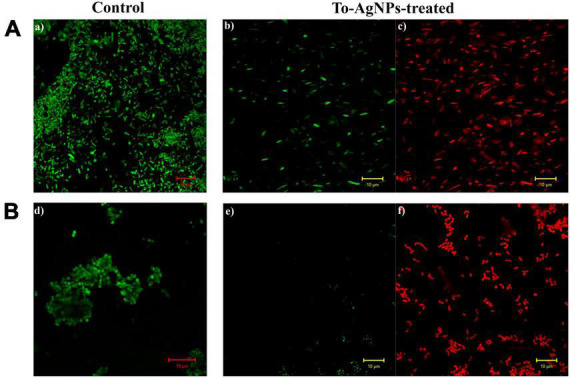
Confocal images of *S. aureus* and *E. coli* stained with acridine orange and ethidium bromide. **(A)** Control and To-AgNPs treated *E. coli.* **(B)** Control and To-AgNPs treated *S. aureus* (green indicates live cells and red indicates dead cells, scale bar 10 μm).

Confocal microscopy analysis of *S. aureus* and *E. coli* treated individually with a lethal dose (MIC) of To-AgNPs revealed the dead and surviving bacteria. In Figure 12, acridine orange and ethidium bromide stainings showed large number of dead cells (red image) and fewer surviving bacteria with physical deterioration and disintegration (green image) in both treated groups, but no dead cells are seen in the control groups.

### 3.11. Scanning electron microscopy (SEM)

**FIGURE 13:**
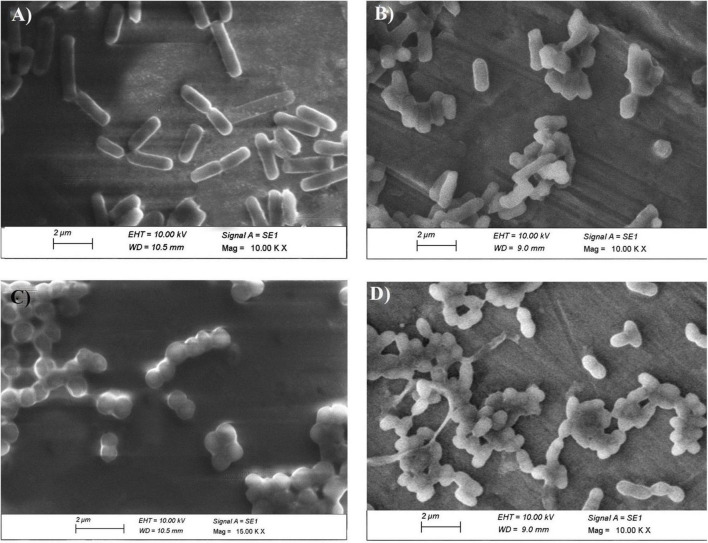
SEM image of To-AgNPs treated and untreated bacteria. **(A)** Untreated *E. coli*, **(B)** To-AgNPs treated *E. coli*, **(C)** Untreated *S. aureus*, **(D)** To-AgNPs treated *S. aureus*.

To further confirm the results observed with CLSM, SEM was performed on the same samples (Figure 13). The untreated *E. coli* cells appeared intact with no signs of ruptures and collapses, while the To-AgNPs-treated *E. coli* cells appeared shorter and compact or completely deformed. For *S. aureus*, the untreated cells appeared as smooth surfaces without any damage to their exterior structures, whereas, the treated cells displayed alterations and deformities of their surfaces and corrugated outer membranes.

## 4. Discussion

Urinary tract infections is one of the most common reasons for the use of antibiotics, which results in the development of antibiotic resistance, a public health concern worldwide. Several investigations are currently conducted with new potential treatments and preventions for UTIs. The antibacterial properties of AgNPs could offer an alternative solution to the resistance issue. It is long-since known that silver-based compounds have antibacterial properties. Exhaustive literatures are available on their antimicrobial, anticancer, and antioxidant activities (Zhang et al., 2020, Algotiml et al., 2022). Using seaweed as a reducing agent in the synthesis of NPs will be beneficial because they are eco-friendly and less biohazardous, and their medicinal compounds can be added to NPs to enhance their performance. No documented studies were done on the antibacterial activities of *T. ornata* extract and its biofabricated nanoparticles against uropathogens. Thus, the present study evaluates the bioactive properties of To-AgNPs as a potential therapeutic agent for UTIs.

### 4.1. To-AgNPs synthesis and characterization

In the present study, an aqueous extract of *T. ornata*, obtained by hot extraction, was used to synthesize AgNPs. Biomolecules present in *T. ornata* function as reducing and capping agents during AgNP synthesis. The addition of 1 M NaOH enhanced the reducing power of functional groups in To-AE and accelerated the formation of small and stable AgNPs. When AgNO_3_ solution was added to the To-AE and the pH was adjusted to 10, there are color changes from dark yellow to reddish brown, resulting from the reduction of Ag^+^ to Ag^0^ due to surface plasmon resonance. Confirmation of AgNPs formation is commonly done by UV-Vis Spectroscopy. The conduction and valence bands in AgNPs are located very close to each other, allowing electrons to move freely. As these electrons oscillate collectively in resonance with the light wave, they generate an SPR absorption band (λ_max_ at 400–420 nm). AgNPs absorb light depending on their size, dielectric medium, and chemical environment (Salleh et al., 2020). The typical band with λ_max_ at 400-420 nm indicates spherical-shaped AgNPs (Raja et al., 2017).

The FTIR spectrum of To-AE and To-AgNPs showed the presence of various functional groups such as flavanoid, phenolic, nitrile carbons and carboxylic acid groups which is also present in AgNPs, indicating the reduction of functional groups during AgNPs synthesis. These functional groups may donate electrons to silver ions, reducing them to silver atoms, which then aggregate to form AgNPs. They also act as a stabilizer by adsorbing onto the surface of the AgNPs and preventing them from aggregating. Researchers observed a similar phenomenon when investigating possible biomolecules responsible for AgNPs synthesized from other seaweed extracts (Krishnan et al., 2015).

The XRD pattern showed sharp Bragg peaks at 38.07°, 43.97°, 64.36°, and 77.32°, indicating the crystalline nature of To-NPs. Some unassigned peaks were also in their vicinity, possibly due to the capping agent that stabilized the nanoparticles. AgNPs synthesized from the seaweed extract of *Enteromorpha compressa* (Ramkumar et al., 2017), also showed similar results.

The shape and size of the AgNPs were clarified by SEM analysis. The morphology of To-AgNPs was clearly seen in the SEM images with an average size of 73.98 nm with diameters ranging from 64.67 to 81.28 nm. In addition, most of the silver nanoparticles produced were spherical, while some were irregular shaped. TEM was also used to determine the size, shape, and crystallinity of To-AgNPs. In agreement with SEM results, most of the particles appear almost spherical on TEM images, while some have irregular shapes. The lighter areas in the image suggest that Ag^0^ is surrounded by bioactive compounds contained in TO-AE. In an experiment using *Sonchus arvensis* leaf extract-mediated AgNPs, Chandraker et al. (2019) observed similar semi-transparent surrounding zones of capping phytochemical constituents.

The DLS measured size was larger than the SEM size since DLS measures the hydrodynamic diameter, which is the diameter of the particle, hydration layer, and other surface ions and molecules that move alongside the AgNPs (Fissan et al., 2014). According to Du et al. (2015), PDI value of <0.5 is preferred for the nanoparticle solutions to be monodispersed. In the present study, a PDI value of 0.313 was obtained, which confirmed the monodispersity of To-AgNPs. The stability of nanoparticles in aqueous solutions can be understood using the zeta potential, a measure of surface charge potential. Nanoparticles with a zeta potential of more than +30 mV or less than –30 mV are said to be particularly stable in the dispersion medium (Erdogan et al., 2019). To-AgNPs’ zeta potential value were found to be –63.3 mV. The results demonstrate that To-AgNPs are negatively charged and more stable, allowing them to maintain their structural integrity over an extended period of time.

### 4.2. Anti-oxidant and anti-uropathogenic analysis of To-AgNPs

Antioxidant and free radical scavenging properties of AgNPs depend on the bioactive compounds present in seaweeds, and they can improve by increasing the concentration of AgNPs. In the present study, the DPPH antioxidant activities of To-AE, To-AgNPs and the standard ascorbic acid were analyzed at various concentrations (10, 20, 340, 60, 80 and 100 μg/ml). It was found that the radical scavenging activity of both To-AE and To-AgNPs were significantly different to those of standard ascorbic acid. To-AgNPs showed higher antioxidant activity (70.09%) compared to To-AE (61.27%). It was also found that, for all groups, there was an increasing trend in radical scavenging activity with concentration. Alharbi et al. (2020) reported that *T. ornata* extract contained excellent antioxidants. Polyphenols, sulfated polysaccharides and terpenoids present in *T. ornata* were probably responsible for the antioxidant properties (Vijayabaskar and Shiyamala, 2012). Chakraborty and Joseph (2016) reported that phenolic compounds in *T. ornata* were the effective antioxidants that were scavenging DPPH free radicals. Our FTIR results also proved the presence of phenolic compounds in To-AE and To-AgNPs that might be responsible for its antioxidant property. Also, AgNPs are intended to transport electrons to the reactive media in order to neutralize the unstable DPPH free radicals (Chandraker et al., 2022a). Prior to the present study were the seaweed *Ecklonia cava* (Venkatesan et al., 2016), *Desmarestia antarctica*, and *Iridaea cordata* (González-Ballesteros et al., 2021) mediated AgNPs studied for their antioxidant activity and provided similar results.

Urinary tract infection-causing bacteria like *P. aeruginosa*, *K. pneumoniae*, *E. faecalis*, *E. coli*, and *S. aureus* were selected for the antimicrobial study of the To-AE and its biofabricated Ag-NPs. Gram-positive *S. aureus* exhibited the greatest zone of inhibition, followed by Gram-negative *E. coli*. A minimum level of activity was found in *P. aeruginosa*. The statistical analysis of antimicrobial activities of To-AgNPs, To-AE, AgNO_3_, DMSO and Cefotaxime showed that To-AgNPs exhibited higher antimicrobial activity than To-AE and AgNO_3_, which is comparable with Cefotaxime. According to Neelamathi and Kannan (2016), the crude methanolic extract of *T. ornata* contains hydrocarbons, acids, aldehydes, ketones, esters, alcohols, halogenated compounds, and aromatics that are likely to be responsible for its antimicrobial properties. It was reported that antimicrobial activity of *T. ornata* extract killed *P. aeruginosa* effectively (Alharbi et al., 2020) and ethanolic extracts were more effective against Gram-positive bacteria than Gram-negative bacteria (Ward and Deyab, 2016). Few reports are published on the synthesis of Sn-O_2_ and Mg(OH)_2_ NPs from *T. ornata* extract has been reported with antibacterial activity (Govindaraju et al., 2020; Sunny et al., 2022). In a study by Anuluxan et al. (2022) using AgNPs derived from *T. ornata* against water-contaminated bacteria, Gram-positive *S. aureus*, *B. circulans*, and *E. faecalis* and Gram-negative *E. coli* and *P. aeruginosa* and found that To-AgNPs demonstrated the strongest antibacterial activity against *S. aureus*, which was consistent with our findings.

The antibacterial effects of To-AgNPs were dose-dependent. Of the five bacterial strains tested, To-AgNPs showed potent activity against *S. aureus* and *E. coli* with MIC values of 0.0625, 0.125 mg/ml, and MBC values of 0.125, 0.25 mg/ml, respectively. There is still a lot of uncertainty about how AgNPs exert their antimicrobial effects. Possibly, the antimicrobial property is due to Ag^+^ released from To-AgNPs, which bind to cellular structural groups (carbonyl, sulfhydryl and phosphate), penetrate into cells, condense DNA and react with proteins (Leng et al., 2020). Ag^+^ ions interact with thiol groups in enzymes and proteins to attack bacteria. Additionally, Ag^+^ ions trigger the release of K^+^ ions in bacteria and target plasma or cytoplasmic membranes. They also interact with nucleic acids, inhibit cell division and prevent bacterial growth. Additionally, the capping agent of the nanoparticle also interacts with the bacterial membrane. All these phenomena lead to damage or even the death of the microorganisms. The results are in agreement with the disk diffusion results. The MIC values from prior research showed a considerable amount of fluctuations in AgNPs’s antimicrobial properties. Consequently, it is difficult to compare the data since there is no accepted method for determining AgNPs’ antibacterial activity (Loo et al., 2018).

### 4.3. SEM and CLSM analysis

The findings from both CLSM and SEM analysis were consistent, with both showing changes in morphology and structure in bacteria, suggesting the affinity of To-AgNPs to attach to the cell surface resulting in cell membrane degradation and deformation. According to our CLSM results, both Gram-negative *E. coli* and Gram-positive *S. aureus* were effectively killed by To-AgNPs treatment. However, *S. aureus* treated with To-AgNPs were almost dead compared to treated *E. coli*. This indicates that To-AgNPs had a greater effect on *S. aureus* than on *E. coli*. The SEM image revealed that the membranes of *S. aureus* were severely damaged and distorted after being exposed to To-AgNPs, resulting in cellular fluid discharge. AgNPs interfered with the permeability of cell membranes and caused mechanical damage by interacting with cell wall components. For *E. coli*, the surviving cells retained their rod shape with smooth surfaces. This is because Gram-positive and Gram-negative bacteria exhibit different AgNPs toxicity due to their structural differences in cell walls. The peptidoglycan layer in Gram-negative *E. coli* is surrounded by two lipid bilayers and lipopolysaccharides that act as antimicrobial barriers, thus preventing silver ions from entering the cell. A permeability barrier formed by the outer membrane diffusion channels and porins in *E. coli* also prevents more To-AgNPs entry. In contrast, Gram-positive bacteria’s cell walls are highly porous and enable the entry of foreign substances into the cell (Lee et al., 2019). These findings offer additional support for the potential and mechanism of action of To-AgNPs to inhibit uropathogens.

Recurrent UTIs may increase the risk of kidney stones. A wide variety of uropathogens may cause infection stones, including *E. coli, S. aureus, P. mirabilis, K. pneumoniae, Streptococcus sp.* and *P. aeruginosa* (Mohan et al., 2022; Raj et al., 2023). Therefore, biofabricated To-AgNPs are promising anti-uropathogenic agents and may act as stone preventers.

## 5. Conclusion

The significant concern for UTI treatment is that traditional antibiotics may not be effective in treating infections caused by antibiotic-resistant bacteria. AgNPs have been found to have low toxicity to human cells and broad spectrum antimicrobial activity, making them a promising alternative to traditional antibiotics. The present *in vitro* study demonstrated that *T. ornata* extracts can be used to reduce and stabilize AgNPs in a fast and environmentally friendly manner. The biosynthesized AgNPs were characterized by UV-Vis, FT-IR, XRD, SEM, TEM and DLS to confirm their formation and stability. The synthesized To-AgNPs were more effective against Gram-positive *S. aureus*, but the Gram-negative *E. coli* was also susceptible to it. The CLSM and SEM analysis also supports this finding. To-AgNPs may therefore be a great alternative to be further researched and developed as an anti-uropathogenic agent due to high biocompatibility, enhanced colloidal stability, and strong antibacterial activity. For the therapeutic use of To-AgNPs, further investigations are needed on the mechanism of antimicrobial activity of AgNPs and the cytotoxic, genotoxic, and inflammatory properties of AgNPs.

## Data availability statement

The original contributions presented in this study are included in the article/Supplementary material, further inquiries can be directed to the corresponding authors.

## Author contributions

CR, RJ, and SK: Conceptualization and resources. CR, SK, and RJ: methodology, analysis, and investigation. RJ and SK: supervision. CR: writing—original draft preparation. SK, KM, RJ, and HD: writing—review and editing. All authors contributed to the article and approved the submitted version.
