# Supplementary figures and images for: Structural characterization, antioxidant and anti-uropathogenic potential of biogenic silver nanoparticles using brown seaweed Turbinaria ornata
Source: Front Microbiol. 2023 Sep 1;14:1072043. doi: 10.3389/fmicb.2023.1072043 (PMC10505674; doi:10.3389/fmicb.2023.1072043)

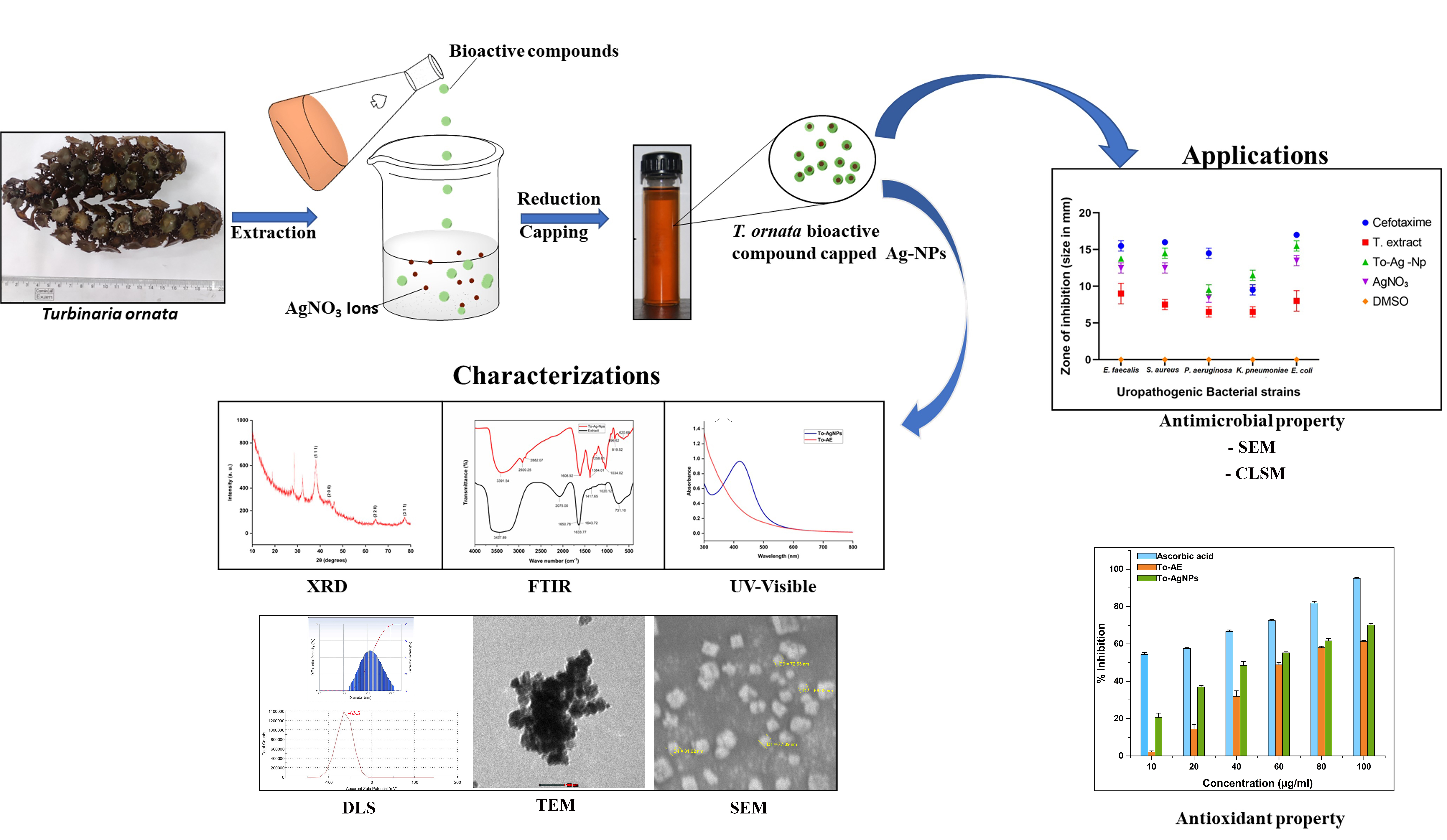

Supplement: Supplementary file 1 [file Image_1.JPEG]

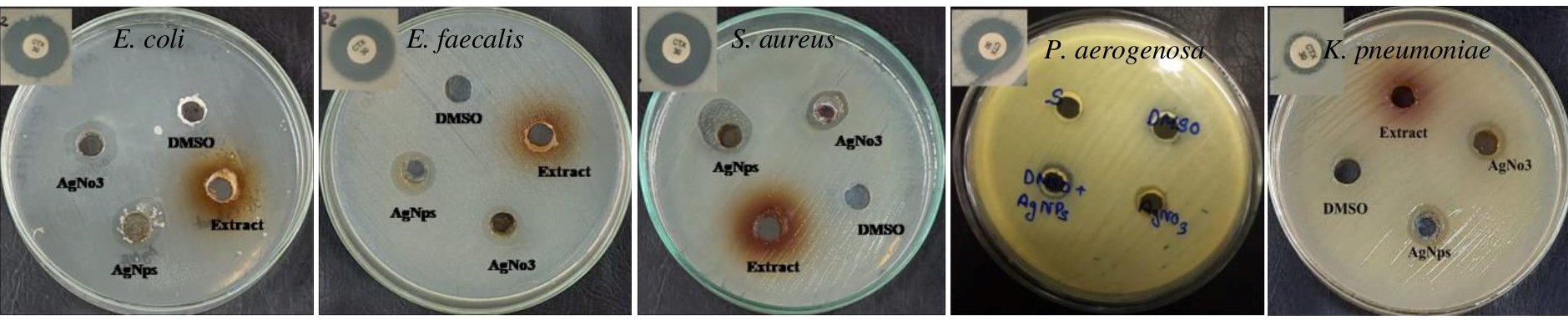

Supplement: Supplementary file 2 [file Image_2.JPEG]
